# A Letter to Dr. Grattan, Containing Some Considerations on His "Remarks on the Importance of the Medical Profession, and on the Present State of Medical Practice in Ireland."

**Published:** 1820-12

**Authors:** Nodes Dickinson

**Affiliations:** Member of the Royal College of Surgeons; Staff-Surgeon to His Majesty's Forces, &c.


FOR THE LONDON MEDICAL AND PHYSICAL JOURNAL.

*A Letter to* Dr. GRATTAN, *containing some Considerations on his "Remarks on the Importance of the Medical Profession, and on the present State of Medical Practice in Ireland."* By NODES DICKINSON, Member of the Royal College of Surgeons; Staff-Surgeon to his Majesty's Forces, &c.

"To improve his profession, both in theory and practice, is the duty, and ought to be the wish, of every professional character: the acquisition of truth should be his principal motive for exertion, and from its pursuit nothing should divert his attention."—GRATTAN'S *Remarks.*

I CONCUR with you in the necessity of attempting to reform the abuses which prevail in the practice of medicine, and of correcting the errors in medical education. These abuses and errors are numerous and important. They have received, indeed, in England a partial correction, by the late Apothecary's Act; but serious errors still remain, demanding reformation.

The announcement of your proposal "to do away some popular prejudices, and succeed in altering opinions erroneously formed," affords peculiar satisfaction. The remedy you suggest, I presume, is derived from a right understanding of the evil which it professes to remove; and it comes more especially recommended to our confidence, "addressed to the public at large" under the seal of collegiate authority.

I have given your "Remarks" an attentive perusal, and I

shall endeavour to appreciate their worth. I shall diligently examine the grounds of your arguments, the evidence of your proofs, and the validity of your conclusions. It will afford me singular gratification to trace in your views the earnest of a disposition actuated solely by motives for the public good; while I confidently anticipate the knowledge that is requisite to a circumspect deduction of consequences, from a consideration of the rank you hold in the profession.

You very properly "think it worth while to examine and expose some of the errors and prejudices that prevent the improvement of medicine;" and you generously set out by disclaiming every selfish and illiberal motive. Your resistance to the application of "several apothecaries and others, practising in the *inferior* departments of the profession, to be admitted as licentiates of the College of Physicians," is founded, you observe, in the opinion that the measure "is inconsistent with *sound* policy, and decidedly opposed to *existing* regulations." Let us, then, briefly consider the grounds of your opposition. Let us endeavour to acquire a just estimate of the soundness of your policy, and of the character of the existing regulations which you are so desirous to uphold.

Permit me, Sir, to ask, does the King's and Queen's College admit any class of associates until possessed of unquestionable evidence of the candidate's abilities and trust? Does the College not require the clearest proof of fitness; and does it not possess the means to determine this important question, by the unexceptional test of an adequate examination? If so, it follows that no unqualified pretender can obtain an entrance into your sanctuary. Every candidate for examination must possess the full measure of merit required, as the ground-work of his reception. Ignorance and imposture are inevitably excluded from an institution thus armed with power to admit or reject at discretion,—thus qualified to appreciate talent and detect incompetency.

To what, then, do this "sound policy," and these "existing regulations," apply? Do they apply to the public good, to the interests of science, or to the security of the private concerns and exclusive privileges of a college or corporation? If the candidate for admission into your College is eligible, under "existing regulations," through *any other* influence than merit fairly ascertained, the policy which permits his reception is erroneous: and if, on the other hand, you refuse the solicitation of a candidate to substantiate his claim to the honour he seeks by a demonstration of his fitness, let the measure be adopted when or where it may, the motive which directs the refusal is founded upon sinister views, inseparable from the selfishness and illiberality you so distinctly disclaim.

You observe, that the abuses which prevail in the practice of medicine have imposed on you the duty to assert its rights and uphold its dignity. That the practice of "the apothecaries and *others*, in the inferior departments of the profession, is in every respect incompetent." "Medicine," you say, "is disgraced by their incapacity, and thousands fall a sacrifice to their want of judgment." This is a serious charge indeed, more especially if your statement be correct, "that innovations on the part of the apothecary, steadily and perseveringly directed to the same object, have at length nearly effected a complete revolution in the state of medical practice." And if thousands fall a sacrifice to this revolution of public opinion in favour of the medical judgment of these "ignorant apothecaries and others," the evil is one that cannot be too deeply deplored.

But if I am to estimate your special objections against this order of men by a consideration of the character and tone of your general reflections, I should hope the enormity of conduct you ascribe to them is less formidable than it is made to appear in the severity of your outline and the depth of your colouring. Perhaps the soundness of policy to which you look up may have a character distinct from the general good, and the regulations you are so determined to maintain may be unworthy to exist.

If the Irish "apothecaries and others," whom you specify to be thus "ignorant and incapable," deserve to rank with their fraternity in England, in their elementary education and professional attainments, which I should take to be the case, from the statement you make of their growing influence with the public, your invectives against them are surely illiberal, and the ground of your aspersions wholly untenable. In a word, if the public gives countenance and support to the "inferior practitioners, the apothecaries and others," whose suit at your bar you deemed it expedient to "resist in every possible way," I should estimate the firmness of their footing on the ground of the public approbation to possess an immovable base. I should be induced to believe these "inferior practitioners," to whom you so violently object, are not so deserving of your ill report, as that you are unhappily influenced in your determination to exclude them from a participation in the practice of medicine by other more cogent reasons than either a conviction of their unfitness, the interests of science, or an anxious solicitude for the public good.

You endeavour to justify the division of the healing art into different departments by the authority of ancient usage; and, in support of your argument thus derived from the sanction of antiquity, you quote a series of truisms, which apply with more than equal force to the opposite side of the question. "When society was in its infancy," you justly remark, diseases were less numerous, and less complicated, than they are at present; and physicians were "exceedingly ignorant." "Individuals, however, acquired a character for extraordinary skill, perhaps in fever or in dropsy;" and thus, as some of them "were more successful than others in the management of particular complaints, medicine was divided into distinct branches, which were practised by different persons:" "a division," you observe, "founded on reason and experience."

The "motive" which you consider "sufficient" for the "division of medicine into different departments," "which was adopted thus early, and has been preserved down to the present day;" which arose at that remote period when "diseases were ascribed to the direct interference of the offended deities," will scarcely be recognized by any scientific practitioner in these comparatively enlightened times.

In the infancy of the healing art, its division, or rather, I should say, its original want of union, arose from the insufficiency and ill arrangement of the facts upon which it is founded. By an extended range of observation and a more accurate investigation of facts, their several relations are better ascertained; the mind is conducted from the mere consideration of individual phenomena, to their various combinations; and, as evidence gradually accumulates, the stock of knowledge at length becomes sufficient to authorize the establishment of general conclusions.

Among the most obvious and inevitable of these conclusions, derived from the clearest testimony of experiment and observation, I am led to consider the impossibility of making a scientific division of diseases into general and local, external and internal; and, although it has been thought expedient to establish practice upon an arbitrary and unnatural division, yet is our daily experience a sufficient proof of the futility of the attempt.

This division of the healing art into different departments, which, you observe, "has withstood the test of ages, and is sanctioned by the authority of antiquity," obtained at a period when those comprehensive views of the animal economy were yet unknown, which in these times have been brought to light chiefly by the aid of physiology founded on the basis of anatomy.

A more intimate acquaintance with the sympathetic relation which subsists between the parts of the living fabric, has in these days enlarged the field of observation concerning morbid actions, and has thus essentially conduced to improve the practice of the healing art. The truth of these positions is attested in various ways. It is clearly ascertained by an attentive consideration of the state of the animal economy, under circumstances in which the doctrine of purely local diseases, as separable in practice from the management of general derangements, must, as a question of medical science, fall to the ground.

These circumstances are particularly marked in the morbid associations which occur in many formidable diseases, originating from obscure and local causes: as, for instance, in certain cases of symptomatic tetanus, in worms, in teething, in cerebral affections produced by derangements of distant organs; in the loss of voluntary power, and in several other nervous disorders, arising from the same cause. In ulcers also, when these are influenced by constitutional affections; and in the latter, when they are either aggravated or subdued by local means. These are a few of the examples which prove the influence of that universal sympathy and association which gives an indivisible unity to the living system, in the collected sum of its organs with the functions they perform, whether healthy or diseased.

So far, indeed, are diseases from preserving their general or local peculiarities, that the most universal disorders occasionally originate in local irritations; while the latter are singularly modified by constitutional influences.

The origin of many general diseases commences in the functional derangement and consecutively-altered structure of the minutest point. This derangement may be confined to a small part of this particular structure, and may be thence removed; or it may traverse every part of the body into which the same structure enters as a constituent: it may, indeed, extend from one structure to another, so as to affect the entire system; or, without producing any obvious derangement of the part to which the morbid cause is directly applied, the diseased action consequent upon such an application may affect the function, or ultimately destroy the structure, of a distant organ, while the original seat and cause of the disorder can only be inferred by a deduction founded on previous experience. It is evident, then, that diseases are so far from preserving their locality with the precision which their separation in practice would require, that it is impossible to define their limits. A bond of connexion is so intimately established between all the parts of the living system, through the inexplicable agency of the vital principle, as to defy every attempt to consider diseases separately and by piecemeal,—general or local, internal or external, exclusively.

"Some diseases," it is true, "are treated *principally* by regimen and internal remedies; while others, on the contrary, are managed, *for the most part*, by mechanical means." But, if we consider in how great a degree mechanical means contribute in the modern practice of surgery, it will afford us no very "sufficient motive" for dividing the healing art into separate departments. It is, moreover, to be remarked, that some constitutional derangements are chiefly remedied by local measures; while certain topical disorders are only assailable by the regulation of diet and internal remedies.

The inference you are so desirous to draw in favour of the physician, "in expressing your sentiments as to the expediency of giving a decided preference to the medical department of the profession," has led you into a strain of peevish discontent, which it would have been more dignified to sustain in silence. It excites a smile to find you so uneasy about those who "stigmatize the physician as incompetent, and represent him as incapable of prescribing with judgment in almost any disease."

In your defence of the *medical* character, you have hazarded certain statements which require a stronger evidence for their proof than mere assertion. It does not seem so clear, by the testimony of experience as you are pleased to represent, that "physicians do recover patients from *confirmed* consumption." The learned and ingenuous practitioner is rather inclined to refer his apparent cures "to the efforts of nature, and not to the interference of the physician," agreeably to this your allusion. He is inclined to express himself with a peculiar diffidence of the powers of art to overcome those inexplicable states of disease, by adopting the confession of inability which you quote, but which you ascribe to "a jealousy and scepticism" of the physician's superior attainments. Indeed, Sir, you plead with an obvious sense of feeling so morbidly acute to the impression of your imagined wrongs, that I fear it is excited by other motives than simply the justice of the cause you undertake to defend.

You observe, "there are some who persuade themselves that the physician, of himself, knows nothing, and that it is to those whom he has been accustomed to *teach* that he must henceforth look for *instruction*." Now it were idle to dwell for a moment on the absurdity implied in the notion of the pupil's becoming his master's teacher. But your statement involves a consequence which, I think, you overlook. Why are "*those* whom the physician has been accustomed to *teach*" so ill instructed by him, "that medicine is disgraced by their incapacity;" that they are "in every respect incompetent?" The public look up to you as their safeguard against the effects of ignorance; the pupil looks up to his instructor for adequate information, in return for the money he pays: both the public and the student are misled, if it be made a boast by the master, that the persons he has been "accustomed to teach" are, notwithstanding his instruction, unqualified to fulfil the duties of their station; and the torrent of obloquy which you pour upon the pupil for his ignorance and presumption, recoils with ten-fold weight upon your own shoulders.

In your "enquiry into the precise merits of the medical profession, strictly so called," you state correctly the proportion of the diseases which you are pleased to denominate "medical;" and you justly observe, that in camps "medical diseases form the majority for which the practitioner is called upon to prescribe." But you omit to notice, that in camps the practitioner is frequently a surgeon, who performs the duty of a physician.. To what effect the military surgeon performs these duties, it were best to ask information from the medical history of the late war, which will sufficiently pronounce upon the merits of naval and army hospital practice.

I admire the pathos with which you describe the "excellence" of your order; "the reputation of those who first practised medicine; the marks of respect which they received during life, and the honours paid to their memory after death:" and I should gladly sympathize with you (p. 16,) in the encomiums even of a Frenchman, whose character is now sufficiently known to determine the value of his praise, although we might suffer by the opinion of his countryman, who was wont to excite on the same subject a very opposite sentiment. In truth, Sir, the value of medical knowledge to the welfare of society, is not to be denied. But the real dignity of the professors of the healing art suffers exceedingly by such appeals as, in your painful anxiety to sustain your imaginary consequence, you have deemed it becoming and expedient to make. Let us be judged, both by the public and among ourselves, rather by our works than by our own conceit. Let us take care to attain a thorough knowledge of the principles and practice of our art. Let us endeavour to deserve the confidence of those who commit themselves to our care: if we succeed in this, we shall then find it easy to set even slander at defiance; we shall not find it necessary to make pathetic appeals in our defence to the public at large; nor shall we be much inclined to place any serious value either on the praises of Rousseau or the satire of Moliere.

"Admission into such a profession as that of medicine, ought not (I grant) to be a mere matter of course." No one should be permitted to practise, until it be ascertained that he has acquired a competent knowledge of the art. On any other grounds, an authority to practise ought not to be conferred. If men are dubbed doctors without a thorough knowledge of the healing art, merely in consequence of a collegiate residence and academic accomplishments, "the name of physician will indeed cease to be an honourable appellation, and become a title desired only by the empty and superficial practitioners, but despised by those who are conscious of real superiority."

You say, most truly, "that study alone will not constitute a physician;" "that theoretical knowledge, when unconnected with practical experience, is dangerous and delusive." Allow me to enquire upon what plan the academic is taught the art of medicine? Is he initiated, before he takes his degree, in the school of "practical experience;" or is he not rather instructed upon the "dangerous and delusive" system you so properly condemn?

There can be no doubt entertained that "previous education is necessary to prepare the mind for the reception of truth:" but, whether the preparative plan of your own education has conduced to this effect, and whether the system adopted by those you are so desirous to crush beneath the weight of your eminence is "that of ignorance which prides itself on its imagined knowledge," remains to be proved.

"If the public do not experience from medicine all the advantages that it professes to afford," still no enquiry into medical qualifications can give them satisfactory information, other than the examinations required by law. The enquiries of the public to ascertain medical merit, are ever liable to the uncertainty which applies to the practice of the art itself, however well conducted, which makes it particularly difficult to appreciate medical talent, even when the investigation is founded upon honourable motives; for neither success nor failure can often justify a decision. It is unfortunate that the only judges of medical merit are those who too often possess an unworthy disposition to depreciate its value.

Your observations on the "boundless extent and almost universal application of chemistry," have already, as I understand, received a particular review: I shall therefore confine myself to an acknowledgment of the happy comparison you have drawn and extended between the admirals and generals with those under their command, to yourself and the apothecaries whom you hold in such sovereign contempt. Nor less striking is the expression of those "feelings of admiration" with which you bestow "the undivided tribute of your praise to the *divine* genius of the architect who designed the classic temple;" after which you very complacently observe, "equal, at least equal, are the pretensions of the physician."

"Ascending in the scale of creation," you advance from chemistry and pharmacy to botany, which I presume is a favourite pursuit, if an opinion may be formed from the vivid style of your introduction to our acquaintance of the "vegetable principle of life which modifies the ordinary play of affinities."

You next inform us, that, "to the physician, mechanical dissection of itself can teach but little;" but that the student "is intimately acquainted with the *general principles* of anatomy." Now, that dissection should teach the physician more than he requires, or "the general principles of anatomy," I will not contend: but that it teaches him thus much, may be safely asserted; as may also, that dissection is the only means by which to attain a knowledge either of the general principles of which you speak, or a knowledge "of the human frame, the order and connexion of the several parts of which it consists," upon which all "general principles" of anatomy must entirely depend.

It is to be hoped that no regular physician is deficient in the knowledge of anatomy. To suppose him ignorant of the structure of that system the injuries of which he undertakes to repair, is to suppose the existence of an error in his education, which surely cannot obtain.*

The want of dissection must keep the physician in a perilous ignorance of the structure and functions of important organs, of the healthy condition of which he cannot be ignorant, and at the same time understand their morbid states. You remark, "an acquaintance with the different parts of the system, so as to explain their general character and mutual relation, is sufficient for the physician, and more than this is completely superfluous." "When we have carried such researches as far as it is possible, what (you ask) is the information that we obtain?" "We arrive at the important discovery that a nerve is a nerve, and are enabled to pronounce, in the language of children, that a muscle is a muscle, because it is one." And on this ground of argument, your physician, "omitting therefore anatomy in its minute detail, learns so much as is really useful and applicable to medical purposes." But, Sir, is it then necessary to inform you, that anatomy without dissection, physiology without anatomy, and pathology without physiology, are "*vox et preterea nihil.*"

It is not so true, that between the laws which regulate the living principle, and those which govern lifeless matter, "there exists no sort of analogy whatever." Nor can you, surely, be ignorant, that every thing we understand concerning "the living principle," the investigation of which you pronounce to be "peculiarly the province of a physician," is entirely derived

* "To what source can we so confidently look for any essential improvements which may be made hereafter in medicine and surgery, as to the more enlightened notions, and more comprehensive views, which may be derived from a further cultivation of anatomy and physiology?"—Brodie's *Introd. Lecture*, 1820.

from our knowledge of the phenomena resulting from function; and that all our knowledge of the " organs, and the manner in which their functions are performed," which you confess is necessary to be understood, is solely derived from patient research into the mechanism of animal structure; a mechanism only to be learned by careful dissection.

When you admit that " as much as is really useful" should be understood of anatomy; that " a certain degree of anatomical knowledge is necessary;" to what particular parts of the wonderful fabric which the physician undertakes to preserve in health and repair in disease, do you restrict the student's investigation? The anatomy of certain organs, you allow, should be understood, in order to explain their functions and to distinguish their diseases: now, considering the intimate relations which subsist in every part of the system, through the inexplicable agency of the vital principle, which parts, what organs, are undeserving attention? What organ, however small and however distant from the chief divisions of the system, may not be the original seat of the most formidable state of general disease? Be assured, Sir, the half-taught anatomist will form only an ill-educated, half-taught, incompetent, and therefore a dangerous, physician.

The student's attention, you say, is occupied with anatomy in so far as to make him " intimately acquainted with its general principles, though he does not devote his entire time to anatomical dissections." To devote one's entire time to any single branch of a complicated art would unquestionably be absurd; but, let me ask you, how you can acquire what you are pleased to designate the " general principles" of anatomy, unless by dissection? " The intimate nature of the principle of vitality," you properly observe, " is amongst those secrets which the human mind never can fully develop: like the principle of gravity, we can only judge of it by its effects." But, then, these effects are only to be understood of the animal body as they are manifested in the phenomena of function and structure.

You observe further on this head, that when anatomy is " principally attended to, it misleads, by occasioning a disposition to theorize on insufficient data." Do you, then, think that, in proportion as our knowledge is profound, we labour under disadvantages from which a superficial attainment is free? Admitting, for instance, that anatomy is less intimately connected than many persons of good judgment consider it to be with a thorough knowledge of the healing art, is it clear to your apprehension that a superficial acquaintance would guard us against a bias to theorize on those insufficient data which you derive from a knowledge more profound? Do you not conceive rather that we ought to refer many of our erroneous conclusions respecting disease to our ignorance of healthy structure, instead of deducing the "incapacity" of a physician from "his superior anatomical knowledge?" To this you answer with a decided negative. "Not only are such studies useless, I am persuaded they are often injurious, and exert a direct influence in rendering the physician of limited practice less competent to prescribe with advantage."

To show that "a minute knowledge of anatomy will not be found to throw much light on the subject of disease," you adduce an improbable, imaginary case. You suppose a most improbable case to occur, in which there is a transposition of all the viscera and vessels; and you then observe, that if disease took place in this anomalous case, the physician, acquainted with the anatomical peculiarity assumed, would be less able to prescribe with accuracy than one who knew nothing of the matter; and from this supposition you finally infer the inutility, and even the disadvantage, of an intimate acquaintance with morbid anatomy. "A case," you remark, "so anomalous *perhaps* never existed:" and what would it prove if founded in fact?

Had it pleased the Creator to make one man entirely different from another in the distribution of his several organs, still, so long as this variety occasioned no error in the performance of the functions, it would be a matter of total indifference to the physician whether his patient was a *right* or *left* hearted man.

But let us take our illustration from a case of frequent occurrence, and reason from facts rather than conjectures on improbabilities. Let us take an example in which the function of an organ is deranged and its structure impaired, and, in consequence of this, other functions and structures are soon drawn into a morbid state: or, to put the case in another way, let us suppose, as often happens, that the organ to which the exciting cause of disease is directly applied does not itself exhibit much appearance of derangement, but that some distant part is principally affected; or that a state of universal disturbance of the system occurs from the slightest topical affection; or, finally, let us contemplate a case in which the function of an organ becomes so gradually deranged as to afford no direct clue, from a consideration of the more obvious general symptoms, to an explanation of the real and original seat of the disease. Reflecting then upon these probable cases of functional and structural derangement, can we venture to refuse the aid of anatomy to direct our diagnosis?

You admit that "morbid anatomy ought not to be entirely overlooked;" which you illustrate by observing, "that the most common and worthless weeds must be described by the botanist, as well as the most valuable plants." That morbid anatomy has been considered, even by its most zealous cultivators, so exclusively the foundation of medical knowledge, as to be "ridiculous and mischievous," requires for its belief a better proof than bare assertion; and so far has it been from depriving us of "all the improvements that have been hitherto made in the theory and practice of medicine," that it has ever contributed to chasten the spirit of self-constituted theories, and to conduct us to the most successful practice.

It is preposterous and absurd to affirm that "the study of morbid anatomy is positively injurious;" and, notwithstanding the quotation from your Report, which you say "contains the grounds of your opinion," I have no doubt the best-informed of the profession will equally refuse their assent to your premises and inference.

Your assertion that "the physician studies the general principles of the living system,—the surgeon its local peculiarities," conveys a meaning more specious than satisfactory. Perhaps, indeed, the surgeons in Ireland merely operate, while the physician directs the medical treatment of the case. If so, although your statement be true, it is such a truth as the surgeon should be ashamed to acknowledge.

Whether this be the case or not, I shall beg leave to remind you, that, among other authorities which are destructive of your arguments "on the propriety of rendering medicine and surgery distinct professions," and on the studies of the physician and surgeon, is the one you have deemed it expedient yourself to quote. Mr. Abernethy sufficiently shows, in his admirable work "on the Constitutional Origin and Treatment of Local Diseases," that, so far are the surgeon's views from being confined to "local peculiarities," as you say, that he *expressly* regards the sphere of his pursuits to embrace the whole consideration of constitutional influences. "No part of the animal body, Mr. Abernethy observes, can in general be very considerably disordered, without occasioning a correspondent derangement in other parts of the system. An evil seems to me to have arisen from the *artificial* division of the healing art into the medical and surgical departments." And, while you confine the study of "the general principles of the living system" to the physician, who alone, you say, "extends his views to the utmost limits of animated nature," you appear to forget that the very authority you quote in support of your opinions, without being of your order, thought it perfectly in character for an accomplished surgeon "to investigate *these universal* laws of vitality," in his Lectures on the general principles of the living system, to the consideration of which you think no head is competent but that of a physician.

I grant that "unless the physician shall have established his future skill on the steady basis of well-directed studies, his practice must ever be defective; but, whether you have "erected the towering column," to which you so beautifully allude, on this steady basis, the public must decide, after a consideration of the plan of education which you have proposed to the student in medicine.

Where an intimate relation subsists among the several departments of any art, so as to establish a reciprocal dependence of one part upon another, as is the case in the art of healing, the reverse of your proposition comes nearest to the truth; and every one must "doubt the advantages which result from these departments being separately cultivated."

The parts which compose a pin, a watch, or other piece of machinery, it is true, may be advantageously constructed by passing through different hands; but then no analogy obtains between the fabrication of these instruments and the preservation and repair of the animal fabric, every part of which is dependent upon every other, and maintained in a state of perfect union and mutual action by the unknown principle of vitality.

I am not aware how the case stands in Dublin, but in London some of the most dignified licentiates of the College of Physicians practise medicine with midwifery. In surgery, the principle you endeavour to defend (p. 44,) is so far from being "admitted and acted upon, from the difficulty experienced by the same person in acquiring a competent knowledge of its different branches," that some of our most eminent surgeons, considering it impossible to form a line of separation, either on the principle of science or on the ground of the public good, practise not only surgery in all its branches, but medicine also; and both of the departments with the greatest honour to themselves, and advantage to society.

There are several surgeons within my knowledge who would not think you paid them the tribute which is due to their acquirements and skill, were you to refuse them their share in the "specific expertness and peculiar tact" with which you affirm that "operations on the large scale is incompatible;" and, notwithstanding your illustration of the subject by a reference to the painter and astronomer, they would not think themselves justified in conceding to you that they are as deficient as you mention with respect to operative surgery on *any* scale; although they might wave their title to the "certain slight of hand" which you deem "so essential to the oculist," (p. 45;) and which is equally so to the cupper and dentist.

If the apothecaries derive their medical knowledge "from observing the practice of different physicians, in consequence of being employed in preparing their recipes," I cannot imagine a worse foundation of skill. But is this really the true state of the case? Does not the prescribing apothecary acquire his knowledge of medicine from the Lectures delivered by *learned physicians* who undertake the instruction of candidates? Do these physicians not give to their pupils certificates of their fitness for examination, under their hands and seals? If the physician, then, is the teacher of medicine to the apothecary; if he receives pay for his tuition, and sends him into society incompetent in such a degree as to authorize the heavy reproaches you heap upon him, however we must lament the ignorance of the pupil, yet what must be thought of the morality of his instructor?

If, however, the apothecary, with all this bad tuition, has "nearly effected a complete revolution in the state of medical practice," as you say: if the public opinion is so far with him that "he prescribes in all cases, and seldom permits a physician to be employed;" who, when employed, "too often conceals the apothecary's mistakes, and consults with him precisely as if he were a regularly-educated practitioner." If this be the state of things, I should be inclined to suspect these apothecaries to have a foundation for this ascendancy over the public opinion, much more in character with "the towering column" of your order than the gross ignorance you impute to them could possibly erect.

You very candidly remark, "In Dublin, notwithstanding all our advantages, we have not hitherto made a conspicuous figure in surgery or pharmacy; nor have we even, since the days of Macbride, produced a single physician whose name will be remembered in future times."

This, Sir, is the most gloomy portrait you have drawn; and you speak of it in a strain of such unhappy feeling, that I am not surprised at the catastrophe summed up in the following declaration. "Matters," say you, "have arrived at such a state in Dublin, and generally throughout Ireland, that it is hardly possible to distinguish the apothecary from the physician." How is this? Is it that the physician has sunk beneath his proper elevation; or is the apothecary rising in the public esteem?

The "surveillance" under which you state the physician to be placed "in the presence of an apothecary at a consultation," is not more extraordinary than it is lamentable to reflect that in these circumstances "the physicians cannot deliberate as freely as they would do, were they by themselves." The reasons you assign for the difficulty in which the physician is placed, and the mode of his escaping it, do not redound to the credit of either party. It is indeed not to be tolerated, that a censor who would press the weight of his criticisms, whether on the science or morality of physic, with a vigour beyond their usual bearing, should affirm that "the presence of an apothecary has so decided an influence over the physician, that, however honest his intentions, he cannot avoid ordering more medicines than he otherwise would:" notwithstanding the opinion he entertains, (and which I consider to be correct,) "that medicine, unnecessarily given, produces a temporary disease, and thus often impedes the efforts of nature, and prevents, instead of promoting, the return of health."

"The history of the rise and progress of every art assures us," as you remark, "that errors and mistakes have at all periods existed, and contributed to retard its advancement." But it is not at all times easy to trace these errors to their legitimate source. In medicine, you seem to think them referable to the encroachments made by the "apothecaries and others" within the precincts of the physician. But if the physician deserves the praises you lavish upon him with so profuse a liberality; if "there is no branch of science or of literature to which they have not contributed more than their proportion; if, in matters immediately connected with the theory or practice of medicine, their works are an imperishable testimony of their industry and talent;" if, in the pursuit of his profession, the physician has considered its improvement both in theory and practice as his principal motive for exertion, unactuated "by a narrow, selfish, corporation spirit, by self-importance, by formality in manners, or by an affection of mystery;"* how are we to account for "that innovation on the part of the apothecary, which," you observe, "has nearly effected a complete revolution in the state of medical practice;" insomuch, you say, "that it is hardly possible to distinguish the apothecary from the physician?" And, if the works of the physicians "are an imperishable testimony of their industry and talent," how is this to be reconciled with the low state of medical science, which, in Dublin, you remark, "has not produced, since the days of Macbride, a single physician whose name will be remembered in future times?"

You observe, that, "if medicine is to return to its former degraded state, at least it shall be said that the causes which have a tendency to produce this change have been pointed out." But I exceedingly doubt the efficiency of the causes to which you ascribe the effect you imagine to exist. I should rather be inclined to refer the elevation of the apothecary in the opinion of the public, to the gradually progressive state of improvement in the healing art; and, although the "supineness" you mention on the part of the physician may have also conduced to support the pretensions of the persevering practitioner in the united departments of medicine and surgery, still the advancement of this order of men to the public confidence, —these " inferior practitioners," whom you so violently attack on the ground of their incapacity, must inevitably be founded on the public opinion, derived from an experience of their ability and trust.

It can never be contended that an ignorant pharmacopolist is a soundly educated physician. But then the wants of society have brought into existence the competent general practitioner: he is duly qualified to practise medicine and surgery; he adds pharmacy to the other branches of the profession, as a mere convenience. With the title of apothecary, or that of general practitioner, he still continues to be paid upon the same plan with the ancient pharmaceutist, who did not prescribe at all. The reform which is principally required in these times, is to ensure, beyond any doubt, the competency of the candidate for general practice to the duties of his station, by the unequivocal test of an examination efficiently conducted; and, when thus authorized, to remunerate him for the exercise of his talent by a proper mode of payment, instead of compelling him to pay himself by a long and disgusting bill for medicines.

The reform here mentioned, in so far as the public are chiefly concerned, is established in England by the Apothecary's Act. The statement you give of the gross ignorance of the Irish apothecaries, appears to me to show the necessity for a similar reform in the sister kingdom. Experience abundantly proves the absolute necessity which has all along existed for the general practitioner to supply the wants of the public. Of the truth of this position no argument is more completely in point, than that the wants of society should have always obliged them to resort for professional aid to those whom you say " disgrace medicine by their incapacity ;" either because the existing medical establishments do not provide the public with competent assistance at a rate of expence they can afford to pay ; or from a disposition in the public themselves to entertain a good opinion of the " inferior" practitioners' abilities, experience, and trust, notwithstanding his deficiency in the proper documents and titles which would impress the seal of authority on his pretension to medical skill.

The preceding pages were written before I saw the review of your opinions which was published in Dublin, or the observations on that review in the second part of your Remarks. On these publications I have no observation to make sufficiently interesting to deserve attention. I am equally convinced with yourself, that, if some change be not adopted by the physicians to secure the public patronage, the apothecary will inevitably continue his professional advice; whether he is encouraged to pursue this lawless measure in consequence of the supineness, or from any oversight on the part of the collegiate professor; or from the sanction of public approbation of his abilities and zeal.*

I concur in your plan either of lowering the terms of your attendance, or of shutting out the incompetent practitioner by supplying your patients with medicines. The only object of medical legislation, is the public good. The public wants must be supplied with the best abilities that can be procured, and at the cheapest cost. None, I think, can refuse their general assent to the physician's skill. I trust also in the efficiency of the well-educated general practitioner, the apothecary of the present day, who is initiated into the profession by the instruction of eminent physicians and surgeons; but I deprecate the remuneration of talent in a liberal art by an unworthy and ridiculous surcharge upon drugs.

Notwithstanding my concurrence in the propriety of your proposal adverted to above, which to me seems well calculated to meet the exigencies of society, I cannot so easily reconcile the inconsistency of your argument against the union of medicine with pharmacy, when I find that, while you affirm the incompatibility of two offices in one person, in order to exclude the apothecary from prescribing, your proposition goes near to make the physician a pharmaceutist, to prevent the apothecary from assuming the duties of the physician.

*London, Wigmore-street; Oct.* 15, 1820.

* "The present system is calculated only for the wealthy, who can afford to pay physicians; or for the wretchedly poor, for whom they prescribe gratuitously. But, for the intermediate and most numerous class, who are neither poor nor rich, no provision is made: they cannot obtain the advice of physicians, and they must, of course, consult apothecaries."—Grattan's *Remarks, Part II.*

